# Feeding a rich diet supplemented with the translation inhibitor cycloheximide decreases lifespan and ovary size in *Drosophila*

**DOI:** 10.1242/bio.061697

**Published:** 2024-11-25

**Authors:** Hye Jin Hwang, Rachel T. Cox

**Affiliations:** ^1^ Department of Biochemistry and Molecular Biology, Uniformed Services University, Bethesda, MD 20814, USA; ^2^ Henry M. Jackson Foundation for the Advancement of Military Medicine, Inc., Bethesda, MD 20817, USA

**Keywords:** *Drosophila*, Cycloheximide, Oogenesis, Protein synthesis, Nutrition, Lifespan

## Abstract

*Drosophila* oogenesis has long been an important model for understanding myriad cellular processes controlling development, RNA biology and patterning. Flies are easily fed drugs to disrupt various molecular pathways. However, this is often done under poor nutrient conditions that adversely affect oogenesis, thus making analysis challenging. Cycloheximide is a widely used compound that binds to and stalls the ribosome, therefore reducing protein synthesis. As egg production is a highly nutrient-dependent process, we developed a method to feed female *Drosophila* a rich diet of yeast paste supplemented with cycloheximide to better determine the effect of cycloheximide treatment on oogenesis. We found that flies readily consumed cycloheximide-supplemented yeast paste. Males and females had reduced lifespans when maintained on cycloheximide, with males exhibiting a dose-dependent decrease. Although females did not exhibit decreased egg laying, their ovaries were smaller and the number of progeny reduced, indicating substandard egg quality. Finally, females fed cycloheximide had disrupted oogenesis, with smaller ovaries, missing ovariole stages, and an increase in apoptotic follicles. Together, these data support that reduced protein synthesis adversely affects oogenesis with a rich diet that provides optimal nutrient conditions. In addition, this method could be used more broadly to test the effect of other drugs on *Drosophila* oogenesis without the confounding effects caused by poor nutrition.

## Teaser

**Summary:** Wet yeast paste supplemented with cycloheximide, a translation inhibitor, affects *Drosophila* oogenesis and reduces progeny number. Using this feeding method circumvents confounding effects on oogenesis due to a poor diet.

## INTRODUCTION

**Fig. 1.**
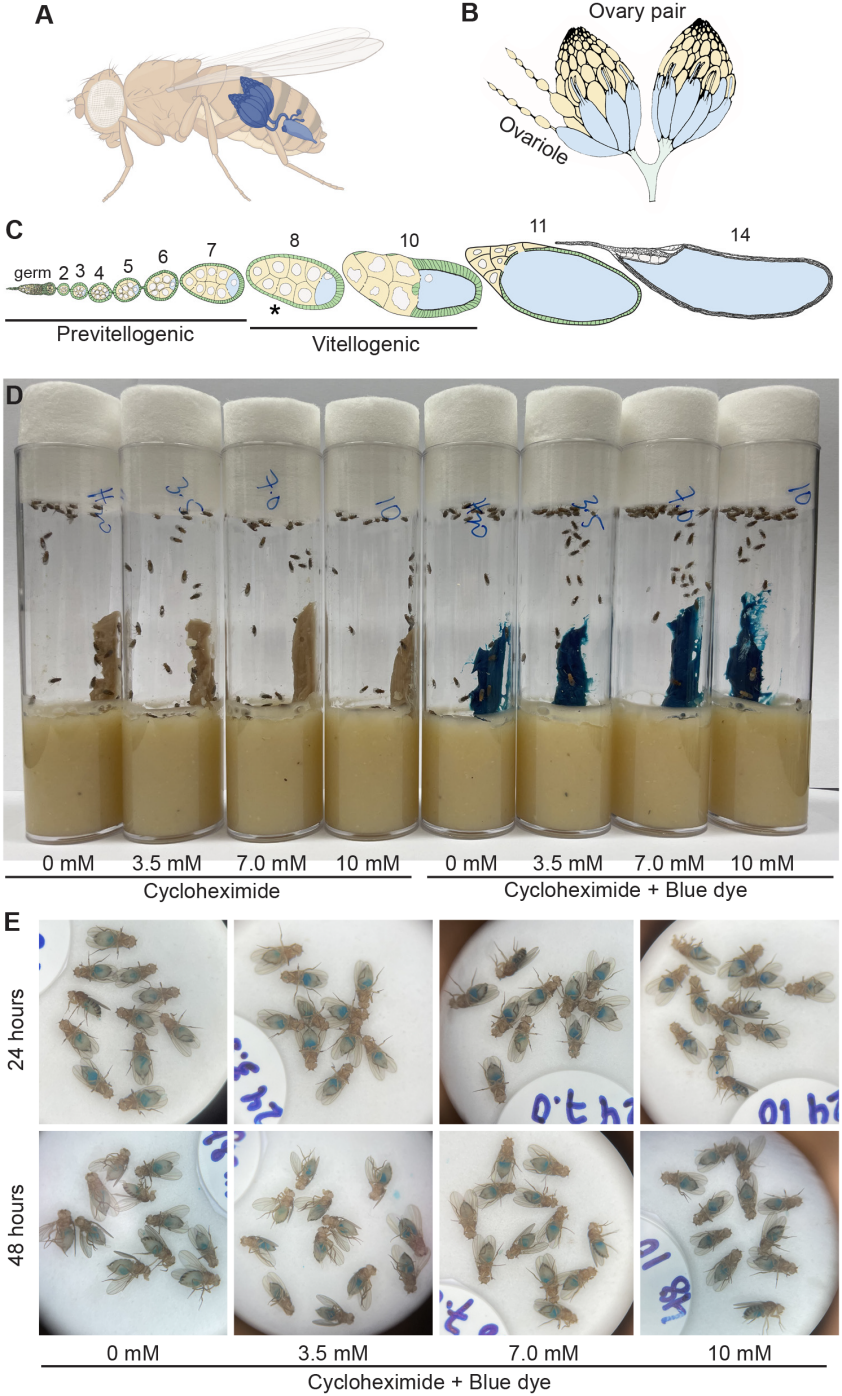
**Flies readily consume wet yeast paste supplemented with CHX.** (A-C) Schematics of *Drosophila* female ovaries. (A) Female flies contain a pair of ovaries. (B) Each ovary is composed of strings of developing follicles called ovarioles. (C) Ovarioles in females fed rich diets contain all stages of follicles (stages 2-14). Stage 8 (asterisk) is a developmental stage that undergoes apoptosis with nutritional stress. (D) Vials of wild-type flies feeding on wet yeast paste with 0, 3.5, 7.0 and 10 mM cycloheximide (CHX). The dye FD&C Blue No. 1 was used to visualize food ingestion by the flies (right). (E) Blue food was visible in the guts of female flies from the vials in D. Thirty animals were tested for each treatment on the same day.

*Drosophila* has been an excellent model system to dissect the genetic pathways and molecular mechanisms controlling processes as diverse as behavior, development and cell differentiation. Females contain a pair of ovaries made up of ∼20 ovarioles (Fig. 1A,B). Each ovariole is a string of developing follicles, with a specialized structure at the anterior called the germarium, which houses the germline stem cells (Fig. 1C). Each follicle is composed of 16 interconnected cells: one cell becomes the oocyte, whereas the other fifteen develop as nurse cells (Fig. 1C) (Spradling, 1993). Oogenesis is energy dependent and highly sensitive to nutrition (Armstrong, 2020). Females fed a diet supplemented with wet yeast paste have maximum germline stem cell division and egg production, with all stages of follicle development present (Fig. 1C). However, when females are fed a poor diet, oogenesis is quickly affected to conserve energy. Clear indications of nutritional stress are apoptosis that occurs at stage 8 and missing follicle stages, although there are other problems including mitochondrial mislocalization, changes to metabolism and apoptosis in the germarium (Drummond-Barbosa and Spradling, 2001). This is a conundrum for researchers wishing to disrupt various pathways and study the effect on oogenesis by feeding flies various compounds. The most common method of drug feeding is using a sucrose solution or spiking the standard fly medium, neither of which constitutes a rich diet and adversely affects oogenesis (Belozerov et al., 2014; Jeong et al., 2022; Kim et al., 2023; Lagasse et al., 2009; Tully et al., 1994).

To circumvent these problems, we developed a method to feed *Drosophila* a rich diet of yeast paste supplemented with cycloheximide (CHX), a translation inhibitor. CHX is a popular compound widely used to study intracellular events and physiological effects by reducing protein synthesis (Darvishi and Woldemichael, 2016; Jevtov et al., 2015; Kim et al., 2023; Lin et al., 2008; Moutaoufik et al., 2014; Sheth and Parker, 2003). This method would allow examining how decreased translation and ribosome stalling affects lifespan and oogenesis without the confounding effects of decreased nutrition. Published articles examining the effects of CHX have used food with limited sources of nutrients when flies were fed with drugs (Kim et al., 2023; Lagasse et al., 2009; Lee et al., 2018; Tully et al., 1994). To support that this feeding method is effective, females fed yeast paste with CHX must readily and reliably consume the drug and exhibit deleterious effects on the fly. Here, we show that feeding CHX in a rich diet reduces lifespan in males and females and females have reduced protein synthesis in the ovary. We also show that feeding CHX using this method does not affect the number of eggs laid but does affect their quality, resulting in reduced number of progeny. In addition, ovary size is reduced and there is an increased amount of apoptotic and missing follicle stages. Overall, our data support that CHX treatment directly reduces lifespan and disrupts oogenesis in flies supported with a rich diet. In addition, this method could also be used to feed other water-soluble drugs to *Drosophila*, provided that the drug is not repellent to the fly, in order to test the effects on development and lifespan without the added stress of a poor diet.

## RESULTS

### Flies readily consume wet yeast containing CHX, decreasing protein synthesis

**Fig. 2.**
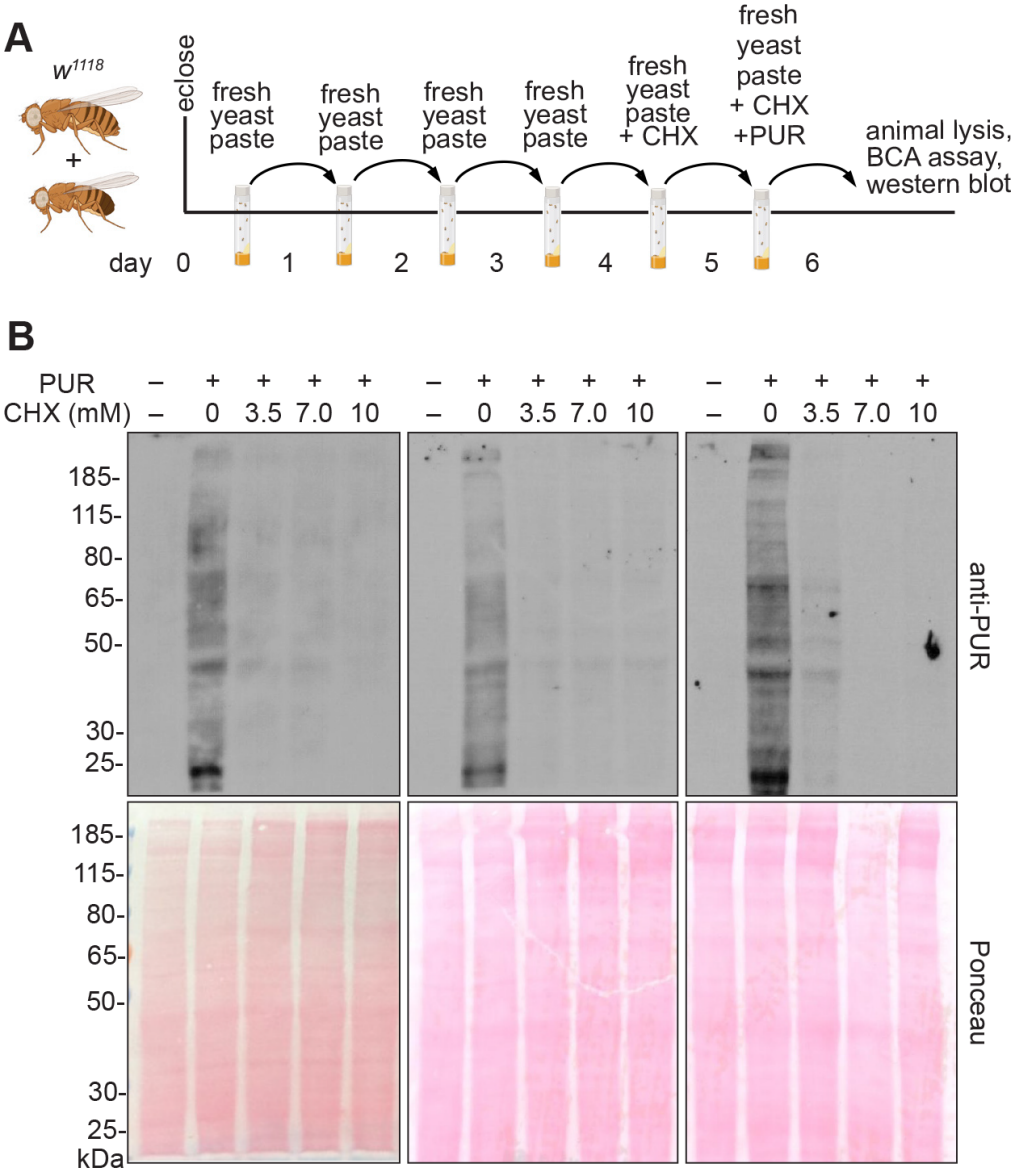
**Flies fed CHX-supplemented yeast paste have reduced protein synthesis.** (A) Schematic illustrating the feeding regimen for puromycin (PUR) incorporation in B. Newly eclosed flies fed freshly prepared yeast paste for 4 days were given fresh yeast paste containing cycloheximide (CHX) for 24 h, after which they were transferred to the food vial with fresh yeast paste containing both CHX and PUR. 24 h later, protein was extracted from the female adults and analyzed. (B) Biological triplicate western blots probed with anti-PUR antibodies showing decreased puromycin incorporation with CHX feeding. 40 µg of protein sample was loaded per lane. Ponceau staining (lower blots) were used as a control for gel transfer to the membrane. PUR –, negative control for puromycin feeding.

To ensure that any CHX effects were due to decreased protein synthesis rather than directly due to poor nutrition that could result from food avoidance by the flies due to drug taste, we fed flies varying concentrations of CHX mixed in their preferred food source: wet yeast paste. To test whether flies would accept and consume this source of CHX, they were fed freshly made CHX-supplemented yeast paste containing increasing concentrations of CHX for 24 or 48 h. We supplemented the food with the dye FD&C Blue No. 1 to track food ingestion (Fig. 1D). Females readily ate the yeast paste as indicated by the blue guts visible in their abdomens (Fig. 1E). To ensure that CHX was having the desired effect and suppressing protein synthesis, we coupled CHX feeding with puromycin (Fig. 2A). Puromycin is a naturally occurring aminonucleoside that mimics tyrosyl-tRNA and incorporates into nascent polypeptides. As it is not amino acid specific, incorporation terminates all translation. Well-fed flies were fed CHX-containing fresh yeast paste for 24 h, then transferred to fresh yeast paste containing both CHX and puromycin and allowed to feed for an additional 24 h (Fig. 2A). Fly extracts were then probed with anti-puromycin antibodies that recognize the terminated nascent peptides (Fig. 2B, upper panels). If CHX decreased translation, we would expect a decrease in the amount of puromycin incorporation. Flies fed 3.5, 7.0 and 10 mM CHX had decreased puromycin incorporation, supporting that this method of feeding CHX is effective (Fig. 2B).

### CHX in a rich diet shortens lifespan and depresses larval growth

**Fig. 3.**
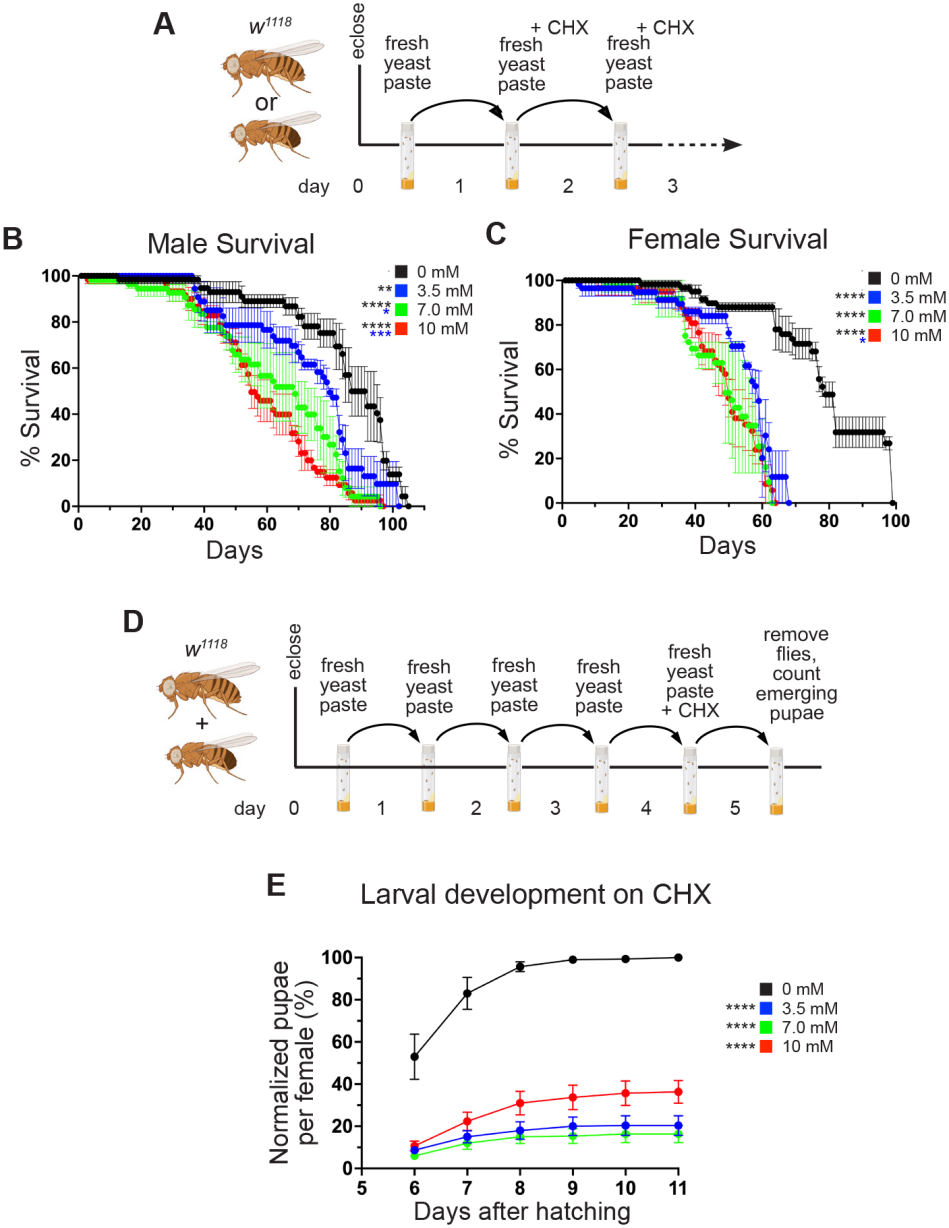
**A rich diet supplemented with CHX affects lifespan and larval development.** (A) Schematic illustrating the feeding regimen for lifespan measurement in B,C. Newly eclosed flies were fed freshly prepared yeast paste for 1 day, after which they were transferred daily to fresh yeast paste containing CHX until they died. (B,C) Lifespan of flies fed with yeast paste containing varying concentrations of CHX at room temperature. Both males (B) and females (C) had significantly shortened lifespans when fed with CHX compared to controls. Males also showed significantly shortened lifespans with increasing CHX concentration. (D) Schematic illustrating the feeding regimen for larval development with CHX in E. Newly eclosed flies were fed freshly prepared yeast paste for 4 days, after which they were fed with fresh yeast paste containing CHX for 24 h, then removed. The progeny from the eggs laid for 24 h were maintained until the pupae emerged. (E) The percentages of pupae per female fed with varying concentrations of CHX normalized to wild type are shown. CHX-fed progenies had significant growth defects compared to the control group. All experimental conditions had three replicates. In B,C,E, error bars show the standard error (s.e.m.). Data were analyzed for significant differences using the log-rank test using the OASIS2 online application with combined triplicates (B,C) or using two-way ANOVA followed by Tukey's post hoc test (E). Asterisks in graphs indicate significance as follows: *P<0.05; **P<0.01; ***P<0.001; ****P<0.0001. Asterisks in E indicate significance on day 6 after egg laying. The colors of asterisks in the graphs denote the concentration of the comparison.

We next tested the effect of dietary CHX on lifespan (Fig. 3A). It is possible that CHX treatment would be quickly lethal to flies if our feeding regimen immediately blocked global translation. However, we found that the flies tolerated daily dietary CHX when fed freshly made yeast paste supplemented with CHX (Fig. 3B,C). We found that continually feeding yeast paste supplemented with 3.5, 7.0 or 10 mM CHX was not immediately lethal but did significantly decrease lifespan in males and females (Fig. 3B,C). Males exhibited a dose-dependent decrease in lifespan, whereas females did not (Fig. 3B versus Fig. 3C). We noticed that the larvae, which hatched from the eggs laid on CHX-supplemented yeast paste, appeared small and delayed. To test whether larvae that grew on CHX-supplemented yeast had delayed development, we counted how many pupae developed (Fig. 3D). CHX strongly suppressed pupation levels compared to those for larvae not reared on CHX (Fig. 3E). These data show that constant exposure to CHX in a nutrient-rich diet decreases lifespan and that larvae exposed to CHX struggle to pupate.

### Females fed a rich diet with CHX have reduced egg quality but not quantity

**Fig. 4.**
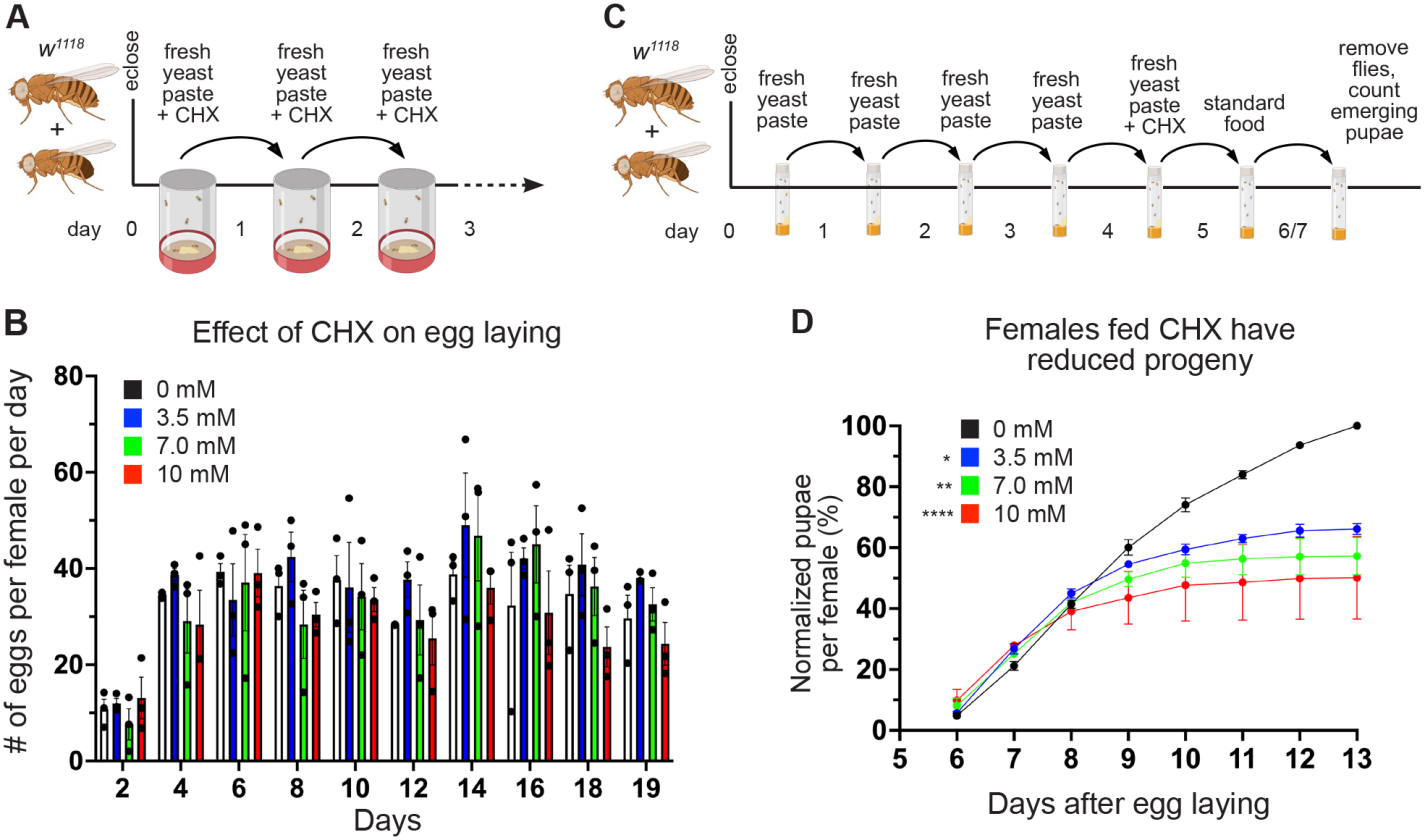
**A rich diet supplemented with CHX affects egg quality but not quantity.** (A) Schematic illustrating the feeding regimen for egg laying measurements in B. Newly eclosed flies were fed freshly prepared yeast paste for 1 day, after which they were transferred daily to an egg-laying cup with fresh yeast paste containing CHX. (B) Quantification of the number of eggs laid per female fed with varying concentrations of CHX. The total number of eggs was counted every 2 days. Points on the graph represent each of three replicates. Bars on the graph represent the average of all three replicates. (C) Schematic illustrating the feeding regimen for progeny developmental assays in D. Newly eclosed flies were fed freshly prepared yeast paste daily for 4 days, after which they were fed fresh yeast paste containing CHX for 24 h. Flies were transferred to a standard food vial and maintained for 48 h, after which they were removed. The progenies from the eggs laid for 48 h were maintained until pupae emerged. (D) The normalized percentages of pupae per female fed with varying concentrations of CHX for 24 h are shown. The number of pupae from flies fed with CHX-containing yeast paste was significantly decreased compared to controls. All experimental conditions had three replicates. In B,D, error bars show the s.e.m. Data were analyzed for significant differences by two-way ANOVA followed by Tukey's post hoc test. Asterisks in graphs indicate significance on day 11 after egg laying. *P<0.05; **P<0.01; ****P<0.0001. The colors of asterisks in the graphs denote the concentration of the comparison.

Once we established that feeding the flies a rich diet with CHX was having an effect, we wanted to determine how decreased protein synthesis affects oogenesis with optimal nutrition. We found that egg laying was not affected, even at the highest dose of CHX (10 mM) (Fig. 4A,B). To test the quality of the eggs laid, we again performed a pupation assay but transferred the CHX-fed females to a standard food vial, letting these females lay eggs in the vial without CHX (Fig. 4C). With this assay, fewer larvae pupated from females fed CHX in yeast paste compared to controls, with a dose-dependent effect (Fig. 4D). These data suggest that, although the number of eggs being laid was unaffected with dietary CHX, egg quality was reduced.

### Females fed a rich CHX diet have ovary defects

**Fig. 5.**
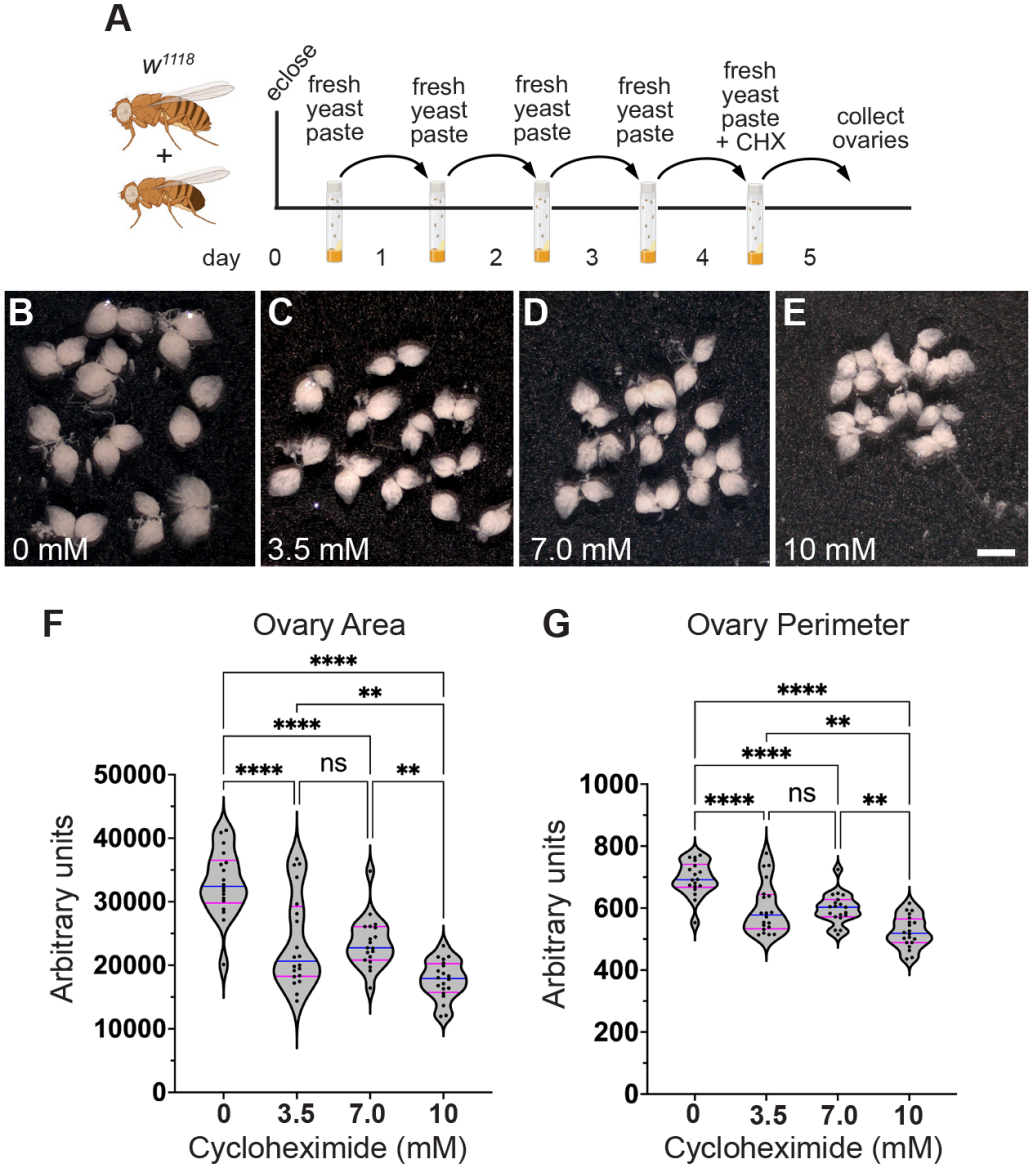
**A rich diet supplemented with CHX affects ovary size.** (A) Schematic illustrating the feeding regimen for measuring ovary size in B-G. Newly eclosed flies were fed freshly prepared yeast paste daily for 4 days, then fed with fresh yeast paste containing CHX for 24 h, after which the ovaries were dissected. (B-E) Ten pairs of ovaries from the females fed with varying concentrations of CHX for 24 h are shown. Females fed CHX had smaller ovaries. The pictures shown are representative of triplicates. These ovaries were analyzed using FIJI/Image J for the whole ovary area (F) and the perimeter (G). Scale bar: 1 mm. (F,G) Violin plots for ovary area (F) and perimeter (G) from females fed CHX. Each point on the violin plot represents one ovary. The median and quartiles are represented by blue and magenta horizontal solid lines, respectively. The plots shown are representative of triplicates. All experimental conditions had three replicates. Data were analyzed for significant differences by one-way ANOVA followed by Tukey's post hoc test. Asterisks in graphs indicates significance as follows: ns, no significance; **P<0.01; ****P<0.0001.

**Fig. 6.**
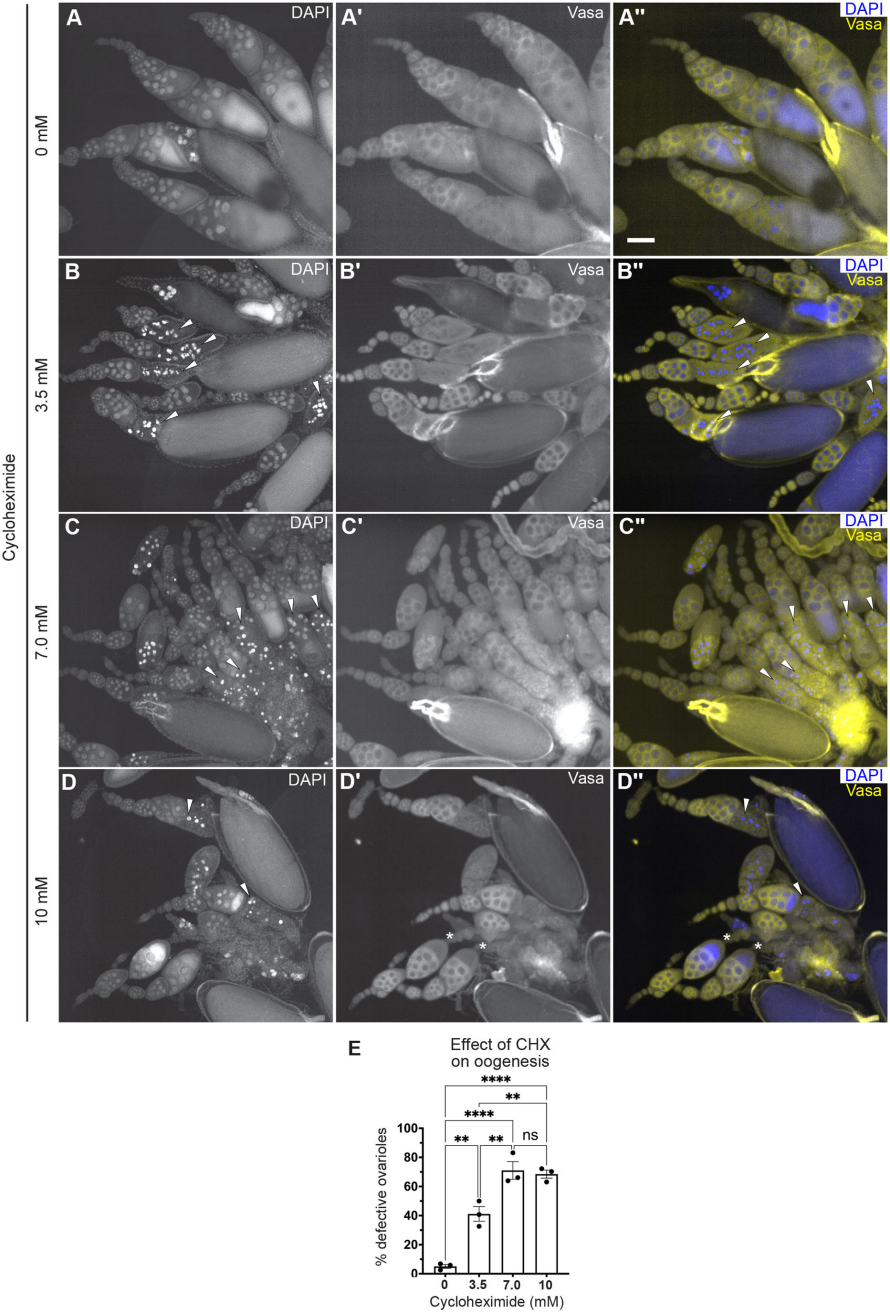
**A rich diet supplemented with CHX affects oogenesis.** (A-D″) Dissected ovaries labeled with anti-Vasa (Aʹ-Dʹ, yellow in A″-D″) to label the germplasm and DAPI (A-D, blue in A″-D″) to label nuclei. Ovaries were dissected from females fed for 24 h with wet yeast paste containing 0 (A-A″), 3.5 (B-B″), 7.0 (C-C″) or 10 (D-D″) mM CHX. White arrowheads indicate apoptotic cells (B-D″). Asterisks indicate ovarioles with missing follicles stages (D′,D″). Ovarioles from CHX-fed females had an increased number of mid-stage apoptotic follicles as indicated by condensed DAPI-labeled nuclei (B,B″,C,C″,D,D″, arrowheads). Scale bar: 100 µm. (E) The relative number of ovarioles having developmental defects shown in A-D″. Ovarioles with developmental defects contained follicles with apoptotic nuclei and/or missing follicle stages. Approximately 50-90 ovarioles were counted in an individual experimental condition (see Materials and Methods for details.) Error bars show the s.e.m. Points on the graph represent each of three replicates. Bars on the graph represent the average of all three independent experiments and data were analyzed for significant differences by one-way ANOVA followed by Tukey's post hoc test. Asterisks in the graph indicate significance as follows: ns, not significant; **P<0.01; ****P<0.0001.

Although egg-laying levels remained the same as the progeny number was reduced, it was possible that there were defects during oogenesis. To identify ovary defects, well-fed females were fed CHX-supplemented yeast paste for 24 h before ovary dissection (Fig. 5A). Whole ovaries dissected from females fed 3.5, 7.0 and 10 mM CHX did not look grossly abnormal but did look slightly smaller compared to controls (Fig. 5B-E). Females fed CHX for 48 h had noticeably smaller ovaries (Fig. S1). To determine any decrease in ovary size after 24 h of feeding, we measured the area and perimeter of each ovary (Fig. 5F,G). Females fed all three doses of CHX had decreased ovary size, with 10 mM CHX causing the smallest ovaries (Fig. 5F,G). Oogenesis is metabolically and energy-intensive and the smaller ovaries might have been caused by less material being made due to reduced protein synthesis. Thus, we extracted and measured total protein from ovaries exposed to CHX. Normalizing for size differences using ovary area, we did not observe a decrease in total protein after 24 h of dietary CHX (Fig. S1G). To determine whether a rich CHX diet altered follicle stage development, we used indirect immunofluorescence. In well-fed females, all stages (2-14) of follicle development in the ovariole are present (Fig. 1C). One of the two developmental checkpoints during oogenesis when follicle death occurs is at stage 8 (Fig. 1C, asterisk). This occurs in response to several stressors, of which the best characterized is nutritional stress (Drummond-Barbosa and Spradling, 2001). To identify any missing follicle stages, we labeled ovaries with the antibody against the germplasm protein Vasa and 4′,6-diamidino-2-phenylindole (DAPI) to label nuclei. Well-fed females had ovarioles that contained the expected follicle stages, with no stage 8 follicles containing condensed nuclei (Fig. 6A-A″). In contrast, females fed a rich diet supplemented with CHX were frequently missing stages and had increased numbers of condensed nuclei, signifying apoptotic follicles (Fig. 6B-D″). Increasing the dose of CHX increased the number of defective ovarioles (Fig. 6E). In the follicles present, we did not detect changes to the number of germ cells per cyst, nor an increase in the loss of the oocyte. These observations support that, with a rich diet, CHX decreases ovary size by increasing apoptotic follicles.

## DISCUSSION

Dietary CHX has been administered to flies in several contexts, but not for its effects on the *Drosophila* ovary and not by feeding a rich diet (Cervantes-Sandoval et al., 2016; Jelen et al., 2023; Jeong et al., 2022; Lagasse et al., 2009; Lee et al., 2018; Takakura et al., 2021; Tully et al., 1994). A previous study examined the effects of CHX feeding on various developmental stages and found a dose-dependent effect on lifespan (Marcos et al., 1982). Behavioral studies have found that CHX feeding affects certain types of memory in flies (Jelen et al., 2023; Lagasse et al., 2009; Lee et al., 2018; Takakura et al., 2021; Tully et al., 1994). These feeding studies used a standard method that relies on a mixture of sucrose and the desired drug and did not use a rich diet, which is critical for supporting optimal ovary development (Drummond-Barbosa and Spradling, 2001; Wei and Lilly, 2014). A sugar-only diet is considered a poor food source for females and does not support robust ovary development and egg laying. As we wanted to observe the effects of CHX on oogenesis without the confounding effects of a poor diet, we fed flies CHX mixed in live yeast paste, a rich diet that *Drosophila* prefer. As the yeast in the paste is active and likely metabolizing the CHX, the paste was made fresh daily. This ensured that CHX levels could be maintained at as steady a level as possible, although there was likely a drop in concentration over 24 h as wet yeast paste contains active yeast cultures that will presumably also metabolize any compound, thus reducing the concentration of drug. Using this method, we saw reduced survival, with females more affected than males. Males, however, exhibited a dose-dependent decrease in lifespan, whereas females did not. Somewhat counterintuitively, female egg laying was not affected. Given that there was increased loss of follicle stages, it is possible that the rich diet combined with stalled translation increased germline stem cell division, making up for any deficit in ovariole development, but we did not test germline stem cell division rates directly. Although egg laying was normal, progeny number was reduced from females fed CHX, indicating that the eggs laid were less competent to develop. The most parsimonious explanation for this observation is that dietary CHX reduces the levels of specific maternal transcripts critical for larval development. Another possibility is that global maternally loaded protein reduction, although not severe, falls below a threshold that cannot support larval development. However, further experiments must be done to determine the specific mechanisms underlying decreased egg competency. Dietary CHX reduced ovary size, likely due to increased apoptosis at a well-known checkpoint, but normalized total protein levels were unchanged. Thus, any action of CHX on disrupting follicle development appears to be independent of decreased global protein levels. Rather, these defects could be due to reduced translation of specific transcripts or signaling pathways while the robust signaling pathways responding to a rich food source remain intact. Overall, these data demonstrate that CHX treatment and stalled ribosomes specifically trigger follicle loss and increase apoptosis. In addition, this feeding method could be used for other compounds to distinguish specific effects on ovary development, as opposed to decreased nutrition.

## MATERIALS and METHODS

### Fly stocks

The fly stock *w^1118^* was maintained on standard cornmeal fly medium. Animals were grown at room temperature. A newly eclosed fly was considered day 0.

### Preparation of CHX solution for wet yeast paste

CHX (Sigma-Aldrich, Burlington, MA, USA; C7698) was dissolved in water to prepare a 10 mM stock solution. Briefly, 140 mg of CHX powder was dissolved in 50 ml of water at room temperature in a dark box by rotating for 30 min, which was aliquoted and stored at −20°C until use. The desired concentration of CHX was prepared by serial dilution of the stock solution. To make fresh yeast paste containing CHX, 0.3 g of active dry yeast (Red Star Yeast) was mixed with 450 µl of CHX solution to create a yeast paste, which was provided daily as needed.

### Blue dye feeding with CHX

Blue dye feeding was performed as described in Wong et al. (2009). Newly eclosed flies were fattened with yeast paste for 3-7 days. Thirty female flies were then transferred to a vial with fresh fly food containing wet yeast paste (control) or freshly made yeast paste with varying concentrations of CHX prepared with 2.5% (w/v) of FD&C Blue No. 1 (Sigma-Aldrich, Burlington, MA, USA; 80717).

### CHX feeding for survival assay

Twenty female or male day 0 adult flies were collected in standard fly food vials containing fresh wet yeast paste. After 24 h, the flies were transferred to freshly made wet yeast paste containing CHX. Fresh yeast paste with CHX was provided daily until no flies survived. Flies were maintained at room temperature, and the dead and missing animals were counted every day. All individual experiments had three replicates. Survival percentage was plotted using GraphPad Prism (GraphPad Software, Boston, MA, USA; https://www.graphpad.com/), and data were analyzed with the Kaplan–Meier test and for significant differences using the log-rank test using the OASIS2 online application with combined triplicates (Han et al., 2016).

### CHX feeding for egg-laying assay

Five newly eclosed female flies and three newly eclosed male flies were collected and fed with wet yeast paste for 24 h in an egg-laying cup containing an agar-corn syrup plate. The flies were transferred to a plate supplemented with wet yeast paste supplemented with varying concentrations of CHX. Every 24 h, the plate was replaced with fresh CHX-supplemented wet yeast paste. The eggs laid on the plate were counted every other day. Each experimental condition had three replicates. The bar graph was generated using GraphPad Prism.

### CHX feeding for pupation assay

Ten female day 0 adult flies and five male day 0 adult flies were collected in a standard food vial with wet yeast paste. Flies were fed yeast paste for 4 days. On day 4, flies were transferred to a standard food vial with wet yeast paste containing CHX. In 24 or 48 h after CHX feeding, flies were transferred to a new standard food vial that was kept at room temperature. After 48 h, flies were removed and the vials were kept at room temperature. The resulting pupae was counted daily and normalized with the number of parent females. All experimental conditions had three biological replicates. The line graph was generated using GraphPad Prism, and the data were analyzed statistically with two-way ANOVA with multiple comparisons followed by Tukey's post hoc test.

### CHX feeding for ovary analysis

Ten female day 0 adult flies and ten male day 0 adult flies were collected in a standard food vial with wet yeast paste. The food vial with fresh wet yeast paste was switched every day for 4 days. On day 4, all female flies in each vial were transferred to a standard food vial containing freshly made yeast paste with varying concentrations of CHX. Fly ovaries were dissected in Grace's Insect Medium (Invitrogen, Waltham, MA, USA; 11595030) 24 h after CHX feeding. To measure whole ovary size, the dissected ovaries were immediately imaged under a stereomicroscope using an Accu-Scope 3076 digital microscope 0.67×-4.5× (Accu-Scope, Commack, NY, USA). After imaging, ovaries were washed with phosphate-buffered saline twice, frozen, and kept at −80°C for protein analysis. Ovary area and perimeter were measured using FIJI/ImageJ (Schindelin et al., 2012). The violin plot was generated using GraphPad Prism, and the data were analyzed statistically using one-way ANOVA with multiple comparisons followed by Tukey's post hoc test. To evaluate CHX effect on oogenesis, the ovaries were immunostained for Vasa and DNA and imaged as described below. Ovarioles were considered having developmental defects if they contained an apoptotic follicle and/or were missing stage 8-11 egg chambers. The following numbers of ovarioles were counted per individual experimental condition: experiment 1–78 (0 mM CHX), 92 (3.5 mM CHX), 97 (7 mM CHX), 72 (10 mM); experiment 2–44 (0 mM CHX), 72 (3.5 mM CHX), 89 (7 mM CHX), 84 (10 mM CHX); experiment 3–68 (0 mM CHX), 81 (3.5 mM CHX), 59 (7 mM CHX), 84 (10 mM CHX). The relative percentage of defective ovarioles compared to normal ovarioles was calculated within each experimental condition. The bar graph was generated by combining the results from three independent experiments using GraphPad Prism, and the data were analyzed statistically using one-way ANOVA with multiple comparisons followed by Tukey's post hoc test. All individual experiments had three biological replicates.

### Puromycin incorporation assay

Fifteen day 0 adult flies and fifteen male day 0 adult flies were collected in a standard food vial with wet yeast paste. The food vial with fresh wet yeast paste was switched every day for 4 days. On day 4, all adult flies in each vial were transferred to a standard food vial containing a wet yeast paste with varying CHX concentration. After 24 h, flies were transferred to a standard vial containing a wet yeast paste with both 600 µM puromycin (Gibco, Waltham, MA, USA; A1113803) and varying CHX concentrations. After feeding puromycin and CHX, the female flies were collected and kept at −80°C until preparing and quantifying protein amounts as described below. 40 µg of each sample was separated by SurePAGE Bis-Tris gel (GenScript, Piscataway, NJ, USA; M00653) and transferred to a nitrocellulose membrane. The nitrocellulose membrane was probed with mouse anti-puromycin (3RH11) antibody (Kerafest, Shirley, MA, USA; EQ0001; 1:2000) following a staining with Ponceau S solution (Sigma-Aldrich; P7170) for imaging of total protein loading.

### Preparation and quantification of cellular proteins

Dissected and frozen ovaries or frozen whole animals were homogenized with a whole-cell lysis buffer composed of 50 mM Tris (pH 8.0), 150 mM sodium chloride, 1 mM ethylenediaminetetraacetic acid (EDTA), 1% NP-40, 0.1% sodium dodecyl sulfate and Complete-mini EDTA-free protease inhibitor (Roche Applied Science, Indianapolis, IN, USA; 11836170001) by repeated freeze-thaw cycles and 30 times of strokes with a pestle. Insoluble material was removed by centrifugation (12,000 ***g*** for 20 min at 4°C) and the subsequent supernatant was collected as a fraction of whole cellular proteins. Protein concentration was determined using Pierce BCA Protein Assay Kit (Thermo Fisher Scientific, Waltham, MA, USA; 23227).

### Immunostaining

Ovaries were dissected with Grace's Insect Medium and fixed for 20 min (4% paraformaldehyde and 20 mM formic acid in Grace's Insect Medium). After washing with antibody wash buffer (0.1% Triton X-100 and 1% BSA in phosphate-buffered saline) three times for 20 min, the tissue was stained with the primary antibody rat anti-Vasa (1:14, Developmental Studies Hybridoma Bank, Iowa City, IA, USA), overnight at 4°C. After washing with antibody wash buffer three times for 20 min, the tissues were stained with the secondary antibody goat anti-rat IgM Cross-Adsorbed Secondary Antibody, DyLight 488 (1:500, Invitrogen, SA510010) overnight at 4°C, then incubated with antibody wash buffer twice for 20 min and stained with 5 ng/ml DAPI solution for 10 min. After removing the DAPI solution, the tissues were mounted in VectaShield Antifade Mounting Medium (Vector Laboratories, Newark, CA, USA; H-1000). Images were obtained using a Zeiss LSM 980 confocal laser scanning microscope at Plan Apochromat 10× objective (Carl Zeiss Microscopy, White Plains, NY, USA) or a Nikon Eclipse Ti2 spinning disk microscope at Plan Apo λ 10× objective (Nikon Corporation, Tokyo, Japan) with *z*-stacks for a depth of 20 µm.

## Supplementary Material

10.1242/biolopen.061697_sup1 Supplementary information

## References

[BIO061697C1] Armstrong, A. R. (2020). Drosophila melanogaster as a model for nutrient regulation of ovarian function. *Reproduction* 159, R69-R82. 10.1530/REP-18-0593 31491744

[BIO061697C2] Belozerov, V. E., Ratkovic, S., Mcneill, H., Hilliker, A. J. and McDermott, J. C. (2014). In vivo interaction proteomics reveal a novel p38 mitogen-activated protein kinase/Rack1 pathway regulating proteostasis in Drosophila muscle. *Mol. Cell. Biol.* 34, 474-484. 10.1128/MCB.00824-13 24277934 PMC3911512

[BIO061697C3] Cervantes-Sandoval, I., Chakraborty, M., MacMullen, C. and Davis, R. L. (2016). Scribble scaffolds a signalosome for active forgetting. *Neuron* 90, 1230-1242. 10.1016/j.neuron.2016.05.010 27263975 PMC4926877

[BIO061697C4] Darvishi, E. and Woldemichael, G. M. (2016). Cycloheximide inhibits actin cytoskeletal dynamics by suppressing signaling via RhoA. *J. Cell. Biochem.* 117, 2886-2898. 10.1002/jcb.25601 27192630

[BIO061697C5] Drummond-Barbosa, D. and Spradling, A. C. (2001). Stem cells and their progeny respond to nutritional changes during Drosophila oogenesis. *Dev. Biol.* 231, 265-278. 10.1006/dbio.2000.0135 11180967

[BIO061697C6] Han, S. K., Lee, D., Lee, H., Kim, D., Son, H. G., Yang, J.-S., Lee, S.-J. V. and Kim, S. (2016). OASIS 2: online application for survival analysis 2 with features for the analysis of maximal lifespan and healthspan in aging research. *Oncotarget* 7, 56147-56152. 10.18632/oncotarget.11269 27528229 PMC5302902

[BIO061697C7] Jelen, M., Musso, P. Y., Junca, P. and Gordon, M. D. (2023). Optogenetic induction of appetitive and aversive taste memories in Drosophila. *eLife* 12, e81535. 10.7554/eLife.81535 37750673 PMC10561975

[BIO061697C8] Jeong, E. M., Kwon, M., Cho, E., Lee, S. H., Kim, H., Kim, E. Y. and Kim, J. K. (2022). Systematic modeling-driven experiments identify distinct molecular clockworks underlying hierarchically organized pacemaker neurons. *Proc. Natl. Acad. Sci. USA* 119, e2113403119. 10.1073/pnas.2113403119 35193959 PMC8872709

[BIO061697C9] Jevtov, I., Zacharogianni, M., Van Oorschot, M. M., Van Zadelhoff, G., Aguilera-Gomez, A., Vuillez, I., Braakman, I., Hafen, E., Stocker, H. and Rabouille, C. (2015). TORC2 mediates the heat stress response in Drosophila by promoting the formation of stress granules. *J. Cell Sci.* 128, 2497-2508. 10.1242/jcs.168724 26054799 PMC4510851

[BIO061697C10] Kim, H. S., Parker, D. J., Hardiman, M. M., Munkácsy, E., Jiang, N., Rogers, A. N., Bai, Y., Brent, C., Mobley, J. A., Austad, S. N. et al. (2023). Early-adulthood spike in protein translation drives aging via juvenile hormone/germline signaling. *Nat. Commun.* 14, 5021. 10.1038/s41467-023-40618-x 37596266 PMC10439225

[BIO061697C11] Lagasse, F., Devaud, J.-M. and Mery, F. (2009). A switch from cycloheximide-resistant consolidated memory to cycloheximide-sensitive reconsolidation and extinction in Drosophila. *J. Neurosci.* 29, 2225-2230. 10.1523/JNEUROSCI.3789-08.2009 19228975 PMC6666331

[BIO061697C12] Lee, P.-T., Lin, G., Lin, W.-W., Diao, F., White, B. H. and Bellen, H. J. (2018). A kinase-dependent feedforward loop affects CREBB stability and long term memory formation. *eLife* 7, e33007. 10.7554/eLife.33007 29473541 PMC5825208

[BIO061697C13] Lin, M.-D., Jiao, X., Grima, D., Newbury, S. F., Kiledjian, M. and Chou, T.-B. (2008). Drosophila processing bodies in oogenesis. *Dev. Biol.* 322, 276-288. 10.1016/j.ydbio.2008.07.033 18708044

[BIO061697C14] Marcos, R., Lloberas, J., Creus, A., Xamena, N. and Cabré, O. (1982). Effect of cycloheximide on different stages of Drosophila melanogaster. *Toxicol. Lett.* 13, 105-112. 10.1016/0378-4274(82)90145-x 6817471

[BIO061697C15] Moutaoufik, M. T., El Fatimy, R., Nassour, H., Gareau, C., Lang, J., Tanguay, R. M., Mazroui, R. and Khandjian, E. W. (2014). UVC-induced stress granules in mammalian cells. *PLoS ONE* 9, e112742. 10.1371/journal.pone.0112742 25409157 PMC4237350

[BIO061697C16] Schindelin, J., Arganda-Carreras, I., Frise, E., Kaynig, V., Longair, M., Pietzsch, T., Preibisch, S., Rueden, C., Saalfeld, S., Schmid, B. et al. (2012). Fiji: an open-source platform for biological-image analysis. *Nat. Methods* 9, 676-682. 10.1038/nmeth.2019 22743772 PMC3855844

[BIO061697C17] Sheth, U. and Parker, R. (2003). Decapping and decay of messenger RNA occur in cytoplasmic processing bodies. *Science* 300, 805-808. 10.1126/science.1082320 12730603 PMC1876714

[BIO061697C18] Spradling, A. C. (1993). Developmental Genetics of Oogenesis. In *The Development of Drosophila melanogaster* (ed. M. Bate and A. M. Arias), pp. 1-70. Cold Spring Harbor: Cold Spring Harbor Laboratory Press.

[BIO061697C19] Takakura, M., Nakagawa, R., Ota, T., Kimura, Y., Ng, M. Y., Alia, A. G., Okuno, H. and Hirano, Y. (2021). Rpd3/CoRest-mediated activity-dependent transcription regulates the flexibility in memory updating in Drosophila. *Nat. Commun.* 12, 628. 10.1038/s41467-021-20898-x 33504795 PMC7840730

[BIO061697C20] Tully, T., Preat, T., Boynton, S. C. and Del Vecchio, M. (1994). Genetic dissection of consolidated memory in Drosophila. *Cell* 79, 35-47. 10.1016/0092-8674(94)90398-0 7923375

[BIO061697C21] Wei, Y. and Lilly, M. A. (2014). The TORC1 inhibitors Nprl2 and Nprl3 mediate an adaptive response to amino-acid starvation in Drosophila. *Cell Death Differ.* 21, 1460-1468. 10.1038/cdd.2014.63 24786828 PMC4131179

[BIO061697C22] Wong, R., Piper, M. D. W., Wertheim, B. and Partridge, L. (2009). Quantification of food intake in Drosophila. *PLoS ONE* 4, e6063. 10.1371/journal.pone.0006063 19557170 PMC2698149

